# Caloric; Its Mechanical, Chemical, and Vital Agencies in the Phenomena of Nature

**Published:** 1844-10

**Authors:** 


### Art. XVIII.

*Caloric; its Mechanical, Chemical, and Vital Agencies in the Phenomena of Nature.* By Samuel Metcalfe, m.d. of Transylvania University.—*London,* 1843. 8vo, pp. 1100.

The nature and objects of this work are by no means sufficiently expressed in its title; for it is nothing less than an attempt to found an entirely new system of physics and physiology, in which caloric plays the part of prime mover, every other agency in nature being subordinate to it. Instead of speaking of it as an "entirely new" system, we should, perhaps, characterize it as a return to the philosophy of the ancients, who, according to our author, had much better notions on this subject than their modern successors; the former referring all the phenomena of nature to one universal and self-active principle,—elementary fire; whilst the attention of the latter is divided amongst various motor forces, such as gravity, electricity, vital force, &c.,—all of which, according to Dr. Metcalfe, are really attributable to this one agency, caloric.

We have every desire to speak well of Dr. Metcalfe's work. It is, we believe, the product of many years of patient and unrewarded study, pursued under many discouragements; and the man who will so devote himself to the extension of our knowledge of nature, deserves well of his race, even if his endeavours are not regarded by others in the same light as he himself views them. But much as we respect our author's industry and zeal, and much as we desire to serve him to the best of our ability, we respect the sacred interests of truth much more; and these compel us to express an opinion of the work far less favorable than we should be most glad to have given. The limited space which any such subject can claim in the pages of this Review, prevents us from entering into such an extended analysis of it, as we should have otherwise been glad to make, with the view of showing our readers the extensive survey of the phenomena of nature which our author has taken, and the merit of the inductions, if true, which he has founded upon them. We are very unwillingly obliged, therefore, to content ourselves with a statement of his general propositions, and with pointing out the errors into which we think that he has fallen.

That no hypothesis on the science of caloric, (or, as Mr. Whewell designates it, *thermotics*,) which has yet been offered to the world, can be regarded by philosophers as satisfactory, is evident from the present unsettled state of opinion upon the subject. Dr. Metcalfe is mistaken, however, in saying that " no one, either among the ancients or moderns, ever attempted to give a regular and systematic history of the mode in which caloric operates in all the molecular and aggregate forces of matter." Such an attempt was made a few years since, by the Rev. Thomas Exley of Bristol; who published a work entitled a 'New Theory of Physics;' wherein all the phenomena of the physical universe at least were traced up to certain attractions and repulsions between the atoms of matter, and certain ethereal atoms; which last seem to us to stand much in the place of Dr. Metcalfe's caloric. Now this work had two great merits, of which our author's is destitute. In the first place, it was the production of an accomplished mathematician, who brought to bear upon his subject powers of reasoning which had been exercised upon the profoundest investigations in regard to quantity and numbers; and we believe we are right in saying, that no true theory of physics is likely to be framed, without the mental habits which this study tends to cultivate; besides which, the very nature of the subject requires that, for its full development, the aid of mathematical processes should be frequently called in. In these points, we think that Dr. Metcalfe's work must be regarded as deficient. His reasoning is far from possessing the closeness and stringency, which the subject particularly requires ; and he makes no attempt whatever to develop his theory in a mathematical form. Moreover we cannot but regard Mr. Exley's plan as superior; inasmuch as he ranks the forces of gravity, electricity, caloric, &c. as the products of one more general agency, which includes them all; instead of erecting any single one of them into *the* power which governs the rest. We apprehend that to the full as good a claim could be set up in behalf of electricity, as the cause of all physical and physiological actions, as that which Dr. Metcalfe endeavours to establish for caloric; and that it would be very easy for a theorist, possessing a tolerable knowledge of these phenomena, to write a very plausible treatise in two goodly volumes, in which this claim should be demonstrated, at least to the satisfaction of its author.

Dr. Metcalfe begins by inquiring into the nature of caloric. That it does not consist (as many have maintained) in mere motion or vibration among the particles of ponderable matter, appears to him sufficiently evident from the following considerations.

" 1. That it may be added to and subtracted from other bodies, and measured with mathematical precision, as all good thermometers demonstrate :

"II. That it augments the volume of bodies, which are again reduced in size by its abstraction.

"III. That it modifies the forms, properties, and conditions of all other bodies, in an endless variety of ways:

"IV. That it passes by radiation, through the most perfect vacuum that can be formed by means of the air-pump, in which it produces the same effects on the thermometer as in the atmosphere.

"V. That it exerts mechanical and chemical forces which nothing can restrain, as in volcanoes, the explosion of gunpowder and other fulminating compounds.

"VI. That it operates in a sensible manner on the nervous system; producing intense pain, and disorganization of the tissues when in excess.

"But if caloric were a mere property or quality, how could it be taken from one body and added to another? Or if it augment the volume of other bodies must it not itself have volume, occupy space, and therefore be a material agent? Would it not be mere jargon, to speak of the radiation, reflexion, connexion and conduction of a mere quality or immaterial property? And if caloric were only the effect of vibratory motion among the particles of ponderable matter, how could it radiate from hot bodies, without the simultaneous transition of the vibratory particles? But it is certain that when iron, copper, and other metals are heated to any temperature below the point of ignition; like boiling water they give off caloric freely, without any sensible loss of ponderable matter." (pp. 5-7.)

Now we do not mean to set ourselves up as advocates of the vibration-theory, or indeed of any other theory of heat; because we conceive that the time is not yet come for erecting such generalizations; but we feel called on to point out what we conceive to be the fallacies of our author's arguments in favour of the *material* theory. The terms *addition* or *subtraction* of heat, as a distinct entity, to or from other bodies, involve a *petitio principii*; since the possibility of such addition or subtraction is the very thing to be proved. Dr. Metcalfe adverts to the experiments which have been relied on, as showing that an unlimited quantity of heat can be generated by friction; and affirms that caloric is not thus produced *de novo*, but that it is merely "forced out of the pores of bodies, in the same manner that water is disengaged from the pores of a sponge by pressure,"—being only liberated so long as the bodies are undergoing condensation. But this explanation appears to us perfectly inapplicable to the celebrated experiment of Sir H. Davy; who found that enough caloric was generated by the rubbing together of two pieces of ice in an exhausted receiver, to occasion their liquefaction. Dr. Metcalfe endeavours to account for this fact by saying that there is an enormous amount of caloric locked up in a state of chemical combination with ice; and that there is no good reason why caloric should not be separated from ice by friction, as well as from other solid bodies. But he forgets that the change produced in the bodies themselves is precisely the reverse of that, which he affirms to be the essential condition of the liberation of caloric in other instances, namely, their solidification; for whilst that change corresponds with the effect of cold, the liquefaction of the ice only takes place ordinarily under the influence of heat. Or, to put the same thing in another form, if there be absolutely more caloric in water at 32°, than in ice, (a position which Dr. Metcalfe would hardly dispute,) whence was the additional caloric obtained? The water could not return to its original condition, without giving out this additional amount. When again frozen, the friction of the ice would again liquefy it; and so on to an unlimited degree. Here, then, appears an unequivocal case of the production of heat, (to use the ordinary phraseology,) by friction, to an *unlimited* extent; a fact quite opposed, as it seems to us, to the *material* theory of caloric.

That the *calorical* state (we beg pardon for coining so uncouth a word, but we cannot find any other to answer our purpose,) of bodies may be measured by thermometers, or may be rendered evident to our senses, is no proof whatever of the *material* nature of heat; since we know very well, that bodies in a state of vibration (*sonorous*, for instance,) will excite a corresponding state in others. Thus when two strings or pipes are tuned to the same pitch, the setting one in vibration will cause the other, if sufficiently near, to emit sound of an equal pitch; and a certain *intensity* of sound will produce a violence of vibration, which occasions very perceptible effects—as when a wine-glass is broken by the mere sound of the voice, on the note to which it reciprocates. It would not be impossible then to construct an acoustic instrument, which should measure both the pitch and intensity of sound, in the same manner as the thermometer measures the intensity of heat. Moreover, that an effect is produced upon our senses by the former agent, is no more a proof that it is itself a material entity, than that the latter is so. Everybody knows that sound is occasioned by the undulations of the sonorous body, which are propagated by other bodies—gaseous, liquid, or solid, to each other, or to our organs of sense. And although we commonly speak of sounds as if they were real existences, capable of addition and subtraction, multiplication and division, yet every scientific man knows that it is not so; and that the intensity of the sound depends upon the extent of the vibrations, and its *pitch* upon their rapidity. Moreover, sound is capable of radiating, being reflected, conducted, &c., just as heat is; the only essential difference being, that we can *trace* the medium through which this takes place in the former case, and cannot in the latter. But will any philosopher venture to assert that we have created a *perfect vacuum*, when we have exhausted the *air* from a receiver by the air-pump; or that *nothing* exists in the vast regions of space, because our atmosphere extends but a limited distance from the earth? We believe that the current of opinion is at present setting quite in the opposite direction; and it is incumbent on Dr. Metcalfe to *prove* the existence of a complete *vacuum*, before he uses the fact of the radiation of heat through it, as an argument for the *material* character of that agent. The champion of the vibratory hypothesis might equally well say "your air-pump merely exhausts *air*, and your experiment only proves that *air* is not the medium of conveying the calorical vibrations; the very fact of the radiation of heat through your so-called vacuum proves the existence of a medium, by which its undulations may be propagated." This would be quite as great an assumption as Dr. Metcalfe's; but an assumption which appears to us equally justifiable, more especially as Dr. Metcalfe himself in maintaining the radiation of heat in a *material* form, through space, virtually declares that there is no such thing as a real vacuum in nature. This, indeed, he often repeats in the course of his treatise; and to us it appears much the same practically, whether space is filled by an ether whose undulations transmit light and heat, or by material particles. In neither case can a *vacuum* be admitted.

To follow Dr. Metcalfe through the chain of reasoning, by which he attempts to prove that the planetary motions are all immediately dependent upon the calorifying power of the sun, and that the laws of motion as propounded (and we had always thought established) by Newton, are no better than ingenious fictions, would be beyond the scope of our criticism; but we must content ourselves with remarking that the arguments upon which the new theory is based, appear to us equally unsatisfactory with the preceding, and that they are as far from overthrowing the received doctrines, as they are from establishing anything preferable in their stead. That the laws of motion and of gravitation may be ultimately combined with other similar *general expressions* of the phenomena of heat, electricity, chemical affinity, &c., and other molecular actions, into some expression of still higher generality, is, we believe, the expectation of most of those philosophers who have given attention to the subject; but this combination will assuredly not involve any subversion of those laws, which, as *general expressions of phenomena* (and no theory can be more) are perfect, and have stood every test that can possibly be devised for detecting their insufficiency or erroneous character. Dr. Metcalfe accuses those who use the word *inertia* as expressing that quality of matter by which it tends to remain in the state in which it is, whether of rest or motion, of a quibble, a play upon words; and thinks that he substitutes something much better when he tells us that "everything in nature is composed of two descriptions of matter—the one essentially active (caloric), and the other passive; as maintained by many of the most distinguished philosophers of ancient Greece," (p. 16;) and that caloric is "a self-acting principle, capable of moving itself, and of generating motion in all other bodies," (p. 12.) That a mass of matter once set in motion will remain so, unless checked by opposing forces, is a physical *fact*, which is not to be contradicted; and the law of inertia, as it is generally termed, is no more than an expression of this fact. On the other hand, that this phenomenon is due, not to a simple property of what we commonly term matter, but to a "self-acting principle" superadded to it, seems to us but a pure hypothesis; to return to which is to revive a mode of philosophizing that has long since been abandoned as totally unproductive of good results as well as fallacious in its character.

The remainder of the first volume is devoted to an exposition of the author's views, as to the connexion of his theory of caloric with the physical sciences in general. In chapter ii, of the First book, we find discussed the atomic constitution of matter, and the relative quantities of caloric in different bodies; and in chapter iii, the forces of caloric in elastic fluids, their expansion and contraction, and the constitution of liquids. In these we find the startling position maintained, that "every description of ponderable matter is actually convertible into light, by a sufficiently intense heat, or by electricity;" which he considers as proved by the following undeniable facts.

"I. That the quantity of light generated by ordinary combustion, friction, or percussion, is always in proportion to the rapidity with which ponderable matter is ignited and volatilized.

"II. That the colour of light thus produced always depends on the species of matter employed.

"III. That the electric spark, (like that produced by the collision of flint and steel,) consists of exceedingly minute portions of ponderable matter in a state of incandescence, as will be shown hereafter by the decisive and beautiful experiments of Fusiniere.

"iv. That when the electric fluid is transmitted through the vacuum of an air-pump, little or no light is produced, as proved by the experiments of De Luc, and afterwards by those of Sir H. Davy.

"v. That the most intense heat of a voltaic battery never produces any light, except when acting on ponderable matter; consequently, that light and heat are not identical, as maintained by some modern theorists; and that neither of them is generated by the mere vibrations of an ethereal medium." (pp. 99-100.)

We are again compelled to express our total dissent from this doctrine; the arguments of Dr. Metcalfe in its favour being, as it seems to us, totally insufficient to establish it; and being in opposition to the well-known fact that, in every case of ignition, combustion, &c., in which light is exhibited, the *matter* of the luminous body is so far from being dissipated or lost, that it may be detected to its full amount either in the body itself or in the new compounds which are formed by the process. Therefore, if there be no loss of ponderable matter, there can be no "conversion" of it into light.

The Second Book contains the application of the same theory of caloric to the phenomena of cohesion, conduction, and radiation—of chemical attraction in general—of solution and of freezing mixtures—and of capillary attraction; with the connexion of molecular with aggregate forces of matter. In many of the views propounded by the author, and in the relations pointed out by him, there is, to say the least, great ingenuity displayed; but we are still compelled, though most unwillingly, to express our conviction that the mode of reasoning which he adopts is very far from being satisfactory. We are continually encountering charges against those who attribute to matter such properties as inertia, attraction, &c.; as having no ground for the assumption of these properties; and as having overlooked the real *cause* of them all, viz., caloric: but the author seems to us to mistake his assumption of this "self-acting principle," which is to account for anything and everything, as a demonstration of its existence; and lays himself open to the sarcasm of Molière fully as much as those at whom he points it, if not more so.

In the Third Book, Dr. Metcalfe attempts to prove the identity of caloric and electricity, or rather, that they are modifications of the same universal element. That the analogies between them, which are ably collected and pointed out by Dr. Metcalfe, are very strong, cannot be denied; but the differences still appear to us stronger, requiring that, for the present at least, their phenomena should be referred to a distinct category. That electrical disturbance should generate heat, and, *vice versâ*, that heat should give rise to electrical disturbance, are facts which may be freely admitted, without the deduction being unavoidable "that if caloric and electricity be not modifications of one power, and the cause of all mechanical and chemical action, the whole of modern science is a perfect chaos of contradictions." (p. 454.) But the analysis of Dr. Metcalfe's arguments on this subject, the ingenuity of which we freely admit, would lead us far astray; and we must content ourselves with a notice of that part of his treatise with which, from its bearing on medical science, we are more immediately concerned.

The Second and larger volume is wholly occupied with the application of Dr. Metcalfe's doctrine of caloric to the phenomena of life; for which, he considers, that this agent satisfactorily accounts. It becomes, in his hands, very much like the vital principle of many physiological writers, capable of so moulding and combining the elements of matter, as to render them capable of performing all the varied and wonderful actions which the animated creation exhibits. Our readers are doubtless familiar with our views on this subject; which we may here briefly recapitulate. We hold it absurd to deny that both physical and chemical powers are at work in the living organism, and have a large share in the production of its actions; but it appears to us equally certain that there is another set of powers also concerned—those to which we give the name of *vital*. These last are exhibited only by organized tissues, possessing a certain chemical composition, and a peculiar arrangement of their structure, which no artificial means can produce; and *in all instances*, the generation of a fabric capable of exhibiting vital phenomena is dependent upon the action of a previously-existing organism. Now the manifestation of these phenomena depends upon certain conditions, the failure of one of which is a total preventive of them. Thus a seed requires for its germination not only heat, but moisture and oxygen; and if the seed have lost its vitality, no amount of these other agents can excite the action in question. It appears, then, that the vital property of the seed, acting under the conditions in question, is the source of the act of germination; or perhaps it might be urged that they are all concurrent causes, equally concerned in producing the effect, because it could not take place in the absence of any one of them. But we cannot single out any one of the physical agents just alluded to as more important than the rest; since germination can no more take place without oxygen or moisture than it can without heat.

Hence we deem it very unphilosophical to assert, as Dr. Metcalfe does, that caloric is *the* efficient cause of the vital actions of living beings; since it is only one of several causes which must concur to produce the results in question. And whilst different actions may be performed under extremely wide varieties of condition, in regard to caloric, oxygen, &c., they all agree in being immediately dependent upon those peculiar properties which an *organized* being can alone furnish. But, according to our author, it is owing to the large amount of caloric which surrounds the elements, at whose expense the living fabric is constructed, that they are capable of uniting into ternary and quaternary compounds, having the aptitude for life. On this we would remark, that the doctrine of "ternary and quaternary compounds," as distinctive of the products of organic chemistry, is now every day becoming less probable; in consequence of the revelations made by the progress of the sciences as to the identity between the laws of affinity which regulate the organic and inorganic products of chemical action. Moreover, one of the principal elements of organic compounds, carbon, has never yet been made to assume any other than the solid form; hence the assumption that it contains a larger quantity of caloric than the metallic bases which Dr. Metcalfe regards as so inert, is quite gratuitous. Besides, Dr. Metcalfe takes no account of those other solid substances which enter, though in small quantity, into some of the most important of all organic compounds; and which seem as necessary to their existence as are the oxygen, hydrogen, and azote, on whose exclusively gaseous form his argument is rested. Thus phosphorus is as essential a constituent of nervous matter as azote or oxygen ; yet we never know it to exist in any other state (when uncombined) than as a solid body; and its combustibility is not greater than that of many of the metallic bases.

That the energy and rapidity of vital action depend in great degree upon the temperature at which it takes place is a fact which is familiar to all physiologists; and in the large amount of facts which Dr. Metcalfe has collected in support of this position, we recognize little or nothing that was not previously well known. That caloric is the immediate and operating source of these actions, however, seems to us to be a mere hypothesis, by no means justified by the premises. We see constant indications in Dr. Metcalfe's book of his tendency to grasp at facts which support his peculiar views, and to leave all others out of consideration. Thus, he deduces from the well-known physiological truth—that the energy of the vital actions of an animal are in proportion to the amount of its respiration—the inference that the caloric generated by the respiratory process is the immediate cause of these actions, and that the generation of caloric is the sole object of the respiratory process. In discussing this part of his subject, he returns to a doctrine which we had imagined to be long since exploded—that the carbonic acid set free by the respiratory process is *generated* in the lungs; thinking it quite a sufficient reply to the doctrine now almost universally received, to urge that if the carbonic acid is formed in the systemic capillaries, the temperature of venous blood ought to be higher than that of arterial blood, the reverse of which is the case. He takes no notice whatever of the experiments of Edwards, Müller, and others, who have shown that carbonic acid continues to be exhaled from the lungs, when the animals are made to breathe hydrogen or nitrogen ; experiments which we have always thought to be far more decisive of this question than experiments on the comparative amount of gases extricable from venous and arterial blood; the results of which will depend rather upon their relative states of combination with the other constituents of the fluid than upon the absolute quantity of each gas. We see no reason why,—on the supposition that the carbonic acid is generated in the system at large, rather than in the vessels of the lungs,—the temperature of venous blood should be higher than that of arterial; for it is quite a mistake to imagine that the union of the carbon and oxygen (the combustion-process) takes place in the *vessels.* It is a process which goes on in the *tissues;* and the blood, circulating through the vessels, contributes to its maintenance by supplying the requisite oxygen, and by removing the carbonic acid produced. The heat generated, therefore, is expended in *keeping up* the temperature of the solids; not in *raising* that of the fluids. That this is the case appears perfectly evident to us, from the consideration of the respiratory process as performed in certain parts of plants in which, there being no circulation of fluid, the tissues themselves act directly upon the air ; and when, owing to the peculiar energy with which changes in their constitution are taking place, the amount of heat generated is very large, and becomes evident to our senses. Dr. Metcalfe passes over altogether, also, the well-ascertained fact, that a large quantity of carbonic acid is set free by the skin. His assertion that arterial blood is hotter than venous rests upon the experiments of Dr. Davy, which he states that he has himself confirmed. We do not object to the experiments themselves, which show a difference of about 3° between the temperature of the left and that of the right ventricle; but we assert that this difference is far from being sufficient to account for the whole quantity of heat generated in the body; for that if the carbonic acid were formed in the lungs, and the heat of the whole body were kept up solely by the combustion in them, the difference would be much greater. This, we think, will be evident, when we bear in mind the large quantity of heat which is being continually removed from the surface by radiation, conduction, and evaporation; a quantity which the aortic stream of blood could not supply, unless it were far hotter than it is. The slight difference in question is fully accounted for, as it seems to us, by the absorption of a quantity of oxygen into the blood, in its passage through the lungs, more than equivalent to the carbonic acid disengaged; a process which is well known to be accompanied by the liberation of caloric. A part of this oxygen may very probably enter into chemical combination with the elements of the blood, producing that difference between venous and arterial fibrin which is well known to exist; whilst another part is probably expended in combining with the sulphur and phosphorus, which are taken in as elements of the food, but which are excreted in the urine in the state of oxygen-acids. Dr. Metcalfe might just as well affirm, as it seems to us, that the conversion of sulphur into sulphuric acid takes place in the kidneys, as that the carbon is converted into carbonic acid in the lungs.

It is with great regret that we find ourselves so continually in collision with Dr. Metcalfe, on the fundamental points of his inquiry; and that we feel it necessary to state, that the errors on which we have felt it our duty to animadvert, pervade more or less the whole remainder of his work. The source of these errors we trace to the constitution of his own mind, which leads him to generalize hastily, and with too little consideration for the facts and arguments which run counter to his views. Yet we can conscientiously recommend the perusal of his work to our readers, as containing a large amount of valuable information, together with many ingenious suggestions and deductions. This is especially the case in regard to the Fifth and Sixth books, which treat of the influence of climate and season on the physical and intellectual condition of the human race; the effects of air, exercise, and aliments; the theory of sleep and of temperaments; of fever, inflammation, hydrophobia, tetanus, and other diseases; and of the *modus operandi* of various remedial agents. Like all enthusiasts and system-makers, Dr. Metcalfe undoubtedly does good, by directing attention to many points which would otherwise escape their due amount of notice. The danger is, lest these should occupy the mind too exclusively, and thus draw off the attention from others, which are of equal or even superior importance. There has probably never been a "system" of any kind, which has not had some truth for its foundation; and the impartial and intelligent reader of Dr. Metcalfe's treatise can feel no doubt, that the foundation of *his* system embodies a vast extent of valuable and undoubted truths. But whilst others of equal value and stability are left altogether out of view, the system erected upon this basis cannot be regarded as secure.

In many parts of his work we have encountered what had the appearance of unreasonable dogmatism; and even of undisguised contempt for the opinions of men whom the world holds in the highest estimation. We feel confident, however, that such expressions are the result of the scientific position which the author has thought himself entitled to assume, rather than of the habitual tone of his mind. Of this, we believe, that the closing paragraphs, with which we must conclude this scanty notice, may be considered as a fair expression.

"And now that I have brought this laborious undertaking to a close, it remains for competent judges to decide how far the principles developed have been founded on a legitimate and comprehensive induction from facts. If true, they must be realized in all the practical concerns of human life, but more especially in improved methods of preserving health and curing diseases. Animated by the grandeur of the subject, and a deep conviction of its vast importance to the welfare of mankind, I have committed myself with unreserved confidence to the guidance of nature, undismayed by the magnitude of the enterprise; believing with Bacon, that in science, as 'in the affairs of civil government, it is better to change many things than one;' and with Sir Edward Bulwer, that 'there does not exist one prejudice which can be called salutary, nor one error beneficial to perpetuate.'

"During the prosecution of this task, I have been often reminded of the deeply-rooted prejudices by which the reformer is surrounded; that the mass of mankind have in all past ages been ungrateful to their best friends; that it is generally a thankless office to oppose opinions long sanctioned by custom, and the authority of distinguished names. To all such admonitions I would reply in the words of Sydenham; that "it is better to assist mankind than to be commended by them;" that if the multitude have been always fond of mysteries, fables, traditions, and quack doctors, it is because their leaders have permitted the great science of nature to remain a sealed book, the profoundest of all mysteries. But when the veil which has so long concealed the beautiful mechanism of the universe shall have been drawn aside, all subordinate mysteries will vanish, and with them a countless multitude of pernicious errors, which have hitherto obstructed every avenue to the temple of wisdom. Nor can there be a rational doubt, that a complete knowledge of the prime Mover would be the perfection of science, and enable us to predict whatever should come to pass in the regular course of nature.

"It must not, however, be supposed, that more than a general outline of this immense subject has been attempted in the foregoing work. Nor does the author presume to flatter himself that he has been free from error. Nor should it be expected that a pioneer of unexplored regions can become so fully acquainted with all their various productions as those who follow, and have more leisure for research into details.

"When the extreme difficulty of the inquiry is duly considered, and the results obtained are contrasted with the previous state of our knowledge; it is hoped that men of enlarged views will be more studious to correct than to censure." (pp. 1098-1100.)

